# Variation in Local and Systemic Pro-Inflammatory Immune Markers of Wild Wood Mice after Anthelmintic Treatment

**DOI:** 10.1093/icb/icz136

**Published:** 2019-08-01

**Authors:** Evelyn C Rynkiewicz, Melanie Clerc, Simon A Babayan, Amy B Pedersen

**Affiliations:** 1 Fashion Institute of Technology, State University of New York, New York, NY 10001, USA; 2 MRC Centre for Inflammation Research, The Queen’s Medical Research Institute, University of Edinburgh, EH16 4TJ, UK; 3 Institute of Biodiversity, Animal Health & Comparative Medicine, University of Glasgow, Glasgow, G12 8QQ, UK; 4 Institute of Evolutionary Biology and Centre for Immunity, Infection and Evolution, School of Biological Sciences, University of Edinburgh, Edinburgh, EH9 3FL, UK

## Abstract

The immune system represents a host’s main defense against infection to parasites and pathogens. In the wild, a host’s response to immune challenges can vary due to physiological condition, demography (age, sex), and coinfection by other parasites or pathogens. These sources of variation, which are intrinsic to natural populations, can significantly impact the strength and type of immune responses elicited after parasite exposure and infection. Importantly, but often neglected, a host’s immune response can also vary within the individual, across tissues and between local and systemic scales. Consequently, how a host responds at each scale may impact its susceptibility to concurrent and subsequent infections. Here we analyzed how characteristics of hosts and their parasite infections drive variation in the pro-inflammatory immune response in wild wood mice (*Apodemus sylvaticus*) at both the local and systemic scale by experimentally manipulating within-host parasite communities through anthelmintic drug treatment. We measured concentrations of the pro-inflammatory cytokine tumor necrosis factor alpha (TNF-α) produced *in vitro* in response to a panel of toll-like receptor agonists at the local (mesenteric lymph nodes [MLNs]) and systemic (spleen) scales of individuals naturally infected with two gastrointestinal parasites, the nematode *Heligmosomoides polygyrus* and the protozoan *Eimeria hungaryensis*. Anthelmintic-treated mice had a 20-fold lower worm burden compared to control mice, as well as a four-fold higher intensity of the non-drug targeted parasite *E. hungaryensis*. Anthelmintic treatment differentially impacted levels of TNF-α expression in males and females at the systemic and local scales, with treated males producing higher, and treated females lower, levels of TNF-α, compared to control mice. Also, TNF-α was affected by host age, at the local scale, with MLN cells of young, treated mice producing higher levels of TNF-α than those of old, treated mice. Using complementary, but distinct, measures of inflammation measured across within-host scales allowed us to better assess the wood mouse immune response to changes in parasite infection dynamics after anthelmintic treatment. This same approach could be used to understand helminth infections and responses to parasite control measures in other systems in order to gain a broader view of how variation impacts the immune response.

## Introduction

Within-host parasite communities can be extremely diverse, with one individual being host to many parasite species simultaneously or sequentially over the course of its lifetime. This is especially true for individuals living in the wild, which face frequent and repeated challenges from both microparasites (e.g., viruses, bacteria, and protozoa) and macroparasites (e.g., helminths and ectoparasites). The composition of these within-host parasite communities can have significant impacts on individual host health, parasite transmission dynamics, and the epidemiology of each parasite species (Poulin 1996; Pedersen and Fenton 2007; Lafferty et al. 2008; Telfer et al. 2008; Johnson et al. 2015; Rynkiewicz et al. 2015). However, while we often consider the host individual as the appropriate unit for investigating within-host processes, for parasites, a host represents a diverse ecosystem with many different habitats, each of which can be infected by different parasites (Griffiths et al. 2014; Restif and Graham 2015; Rynkiewicz et al. 2015). Therefore, a parasite’s preferred niche can be functionally determined by the properties of the tissues it infects and by the type of immune response it elicits (Graham et al. 2007; Pedersen and Fenton 2007; Lello and Hussell 2008). Correspondingly, the host immune system has evolved different mechanisms to respond to infection by a diverse set of parasites (Hajnicka et al. 2005; Baucom and de Roode 2011; Maizels et al. 2012; Parker et al. 2017). The host’s response to parasite infection can be measured at multiple sites or scales, including a local response at the site of infection which may focus on tissue repair or controlling initial parasite spread and damage, and a systemic response across the whole organism where circulating immune factors in blood and the spleen coordinate a more general response to infection. While local immune responses impact host susceptibility and infection success of parasites that infect the same location, systemic responses impact susceptibility to parasites coinfecting other tissues within the host (Mwatha et al. 1998; Munoz et al. 2005; Semenya et al. 2012; Zavala et al. 2018). Therefore, investigating parasite–host and parasite–parasite interactions at multiple within-host scales will improve our ability to measure and predict parasite spread as well as quantify variation among hosts in how they respond to infection.

Coinfection is very common in natural host populations, and in turn can lead to within-host parasite interactions (Poulin 1996, 2001; Abu-Raddad et al. 2006; Seabloom et al. 2015). These can be direct via competition for habitat, or indirect through competition for a nutritional resource or via the host’s immune system (Pedersen and Fenton 2007; Graham 2008; Griffiths et al. 2014; Budischak et al. 2015; Griffiths et al. 2015). An analysis of human coinfection networks showed that coinfecting parasites most commonly interact though shared host resources and a common infection site (Griffiths et al. 2014). However, there was also evidence of immune-mediated parasite interactions, where one parasite can negatively or positively impact the infection success or intensity of another through a shared immune response. When parasites interact via the host immune system, it is important to consider at which within-host scales the interaction is occurring (e.g., are these parasites infecting the same or different tissues) to assess their impact on host health or parasite transmission. For example, parasites that elicit a strong systemic immune response can potentially impact host susceptibility to other coinfecting parasite species, even if they infect different within-host habitats. This type of interaction has been shown in wild African Buffalo, where reducing gastrointestinal nematode burdens using anthelmintic treatment significantly increased individual hosts’ odds of survival after bovine tuberculosis infection, due to altered systemic inflammation (Ezenwa and Jolles 2015). Consequently, the scale at which we measure metrics of the immune response will influence our conclusions about how individuals respond to infection, and how the variation in these responses may impact parasite coinfection and transmission dynamics.

How a host responds to infection can also vary depending on demographic/physiological characteristics such as age, sex, or reproductive condition, which can impact resource allocation to local and systemic immune responses (French et al. 2007; Pedersen and Greives 2008; Vandegrift et al. 2008; Buehler et al. 2009). Studies in Soay sheep and tree swallows, for example, have shown significant changes in a host’s immune response with increasing host age, with older hosts in most cases having higher levels of inflammatory markers (Palacios et al. 2007; Nussey et al. 2012; Cevenini et al. 2013; Babayan et al. 2018b). Furthermore, there are key immunological differences between the sexes, with males often being less immune responsive than females. These differences are often attributed to steroid hormones, such as testosterone, whose immunosuppressive effects can also vary across seasons (Demas and Nelson 1998; Lehmer et al. 2010; Pap et al. 2010). There are also immunological differences within the sexes, dependent on life-history stage. For example, pregnant females often have lower levels of inflammatory markers than non-reproductively active females (Martin et al. 2008; Chatterjee et al. 2014; Gorsich et al. 2014). These cases suggest the physiological trade-offs between sexually-selected traits and the immune response are an evolutionarily adaptive mechanism (Folstad and Karter 1992; Zuk 1996; McKean and Nunney 2005; Stoehr and Kokko 2006), emphasizing the importance of studying the impacts of these trade-offs in evolutionarily- and ecologically-relevant contexts.

One key immunological process that can be measured at both the local and systemic within-host scales is inflammation, broadly defined as the recruitment of immune cells and signaling molecules to the site of an infection (Sompayrac 2012). Inflammation occurs early in an infection, involves both the innate and adaptive arms of the immune system, initiates tissue repair or containment, and/or clearance of an infectious agent. Inflammation is maintained by positive feedbacks, which are then disrupted or deactivated by subsequent regulatory or anti-inflammatory signals upon clearance of infection (Sears et al. 2011). One key cytokine involved in controlling inflammation is tumor necrosis factor alpha (TNF-α), a pro-inflammatory cytokine which can activate both innate and adaptive immune responses (Vassalli 1992; Pasparakis et al. 1996; Graham et al. 2007; Bradley and Jackson 2008; Tumanov et al. 2010). In addition, it has been suggested that TNF-α levels indicate a host’s ability to respond to a broad range of parasites, such as worms, bacteria, viruses, or other microbial pathogens (Smith and Ovington 1996; Goff et al. 2002; Sjöwall et al. 2005; Zavala et al. 2018).

Here, we investigated the interactions between TNF-α as a marker of inflammation and parasite infection and coinfection in wild wood mice (*Apodemus sylvaticus*). Wood mice are commonly infected with a diverse community of parasites (Bown et al. 2006; Behnke et al. 2009; Knowles et al. 2013), including the gastrointestinal nematode *Heligmosomoides polygyrus*, which is closely related to *H. bakeri*, an established laboratory model for human nematode infections (Behnke et al. 2005; Maizels et al. 2004; Reynolds et al. 2012). *Heligmosomoides bakeri* is known to elicit strong immunomodulatory effects on the host’s immune system and skews the host response from a pro-inflammatory (Th1-type) immune profile to an anti-inflammatory (Th2/T_reg_-type) immune profile (Maizels et al. 2009; Maizels et al. 2012). Through the use of anthelmintic treatment, we have shown that in the wild, *H. polygyrus* negatively interacts with another common gastrointestinal parasite of wood mice, the coccidian *Eimeria hungaryensis* (Fenton et al. 2014; Clerc et al. 2019b), and that this strong negative effect of *H. polygyrus* on *E. hungaryensis* infection burden is localized to the small intestine (Knowles et al. 2013). However, we currently lack an understanding of how interactions between *H. polygyrus* and *E. hungaryensis* impact immune responses outside of the local site of infection. In addition, studies that experimentally investigate within-host variation in the immune response across different scales are rare, especially in wild populations. Researchers often generalize host immune reactivity from systemic measures, therefore there may be significant implications for understanding and predicting parasite community interactions at the host individual and population scales given the possible differences in immune activity between the local and systemic scales.

We used the production of the pro-inflammatory cytokine TNF-α in response to a panel of toll-like receptor (TLR) agonists in mouse mesenteric lymph node (MLN) cells as a proxy for the local inflammatory response surrounding the gastrointestinal tract, and in spleen cells as a proxy for the systemic inflammatory response, to elucidate how each immune response may mediate within-host interactions between *H. polygyrus* and *E. hungaryensis*. Furthermore, we used anthelmintic treatment to assess the effects of *H. polygyrus* removal/reduction on inflammation at the local versus systemic scales. We predicted that TNF-α production would differ between local and systemic sites due to different immune cell populations being involved and their proximity to the site of infection (Vassalli 1992). Because *H. polygyrus* modulates the host immune response toward an anti-inflammatory immune profile (Maizels et al. 2012; Wammes et al. 2016), we further hypothesized that anthelmintic-treated mice would have increased TNF-α production because of the reduced worm burden and subsequent reduced immunomodulatory effects. Due to the immunosuppressive effects of testosterone (Saino et al. 1995; Zuk 1996; Muehlenbein and Bribiescas 2005; Nunn et al. 2009), we also predicted that male mice would have lower TNF-α levels than females. Lastly, we expected older hosts to have higher concentrations of TNF-α, in line with the concept of immunosenescence (Cevenini et al. 2013). Even though TNF-α is not thought to be directly protective against *H. polygyrus*, it may be associated with the inflammatory immune response to *E. hungaryensis* infection (Smith et al. 1995; Pakandl et al. 2008; Maizels et al. 2009; Maizels et al. 2012).

## Methods

### Field sampling

We conducted two field experiments in wild wood mouse populations in Callendar Woods, Falkirk, Scotland (55.99°N, 3.77°W). We performed the experiment over two trapping sessions, each session lasting 8 weeks (Session 1: October to November 2014 and Session 2: June to July 2015). In 2014, the experiment consisted of two trapping grids in which two Sherman live traps (H. B. Sherman 2 × 2.5 × 6.5-inch folding trap, Tallahassee, FL, USA) were placed every 10 m in a 130 × 80 m grid (total 256 traps). In 2015, the large grid was split into two smaller grids that were separated by ∼10 m, and an additional third grid was added ∼50 m away from the other two grids. All three grids were 70 × 70 m in size and consisted of a total of 294 traps, leading to similar trapping effort in both years. Traps were baited with cotton bedding, mixed seeds, dried mealworms, and a piece of carrot in the late afternoon before each trapping night. The following morning, traps were checked for the presence of animals and all animals were processed and released at the site of capture. For both of the trapping sessions, we trapped three consecutive nights per week for a total of 24 trap-nights per session (Home Office Project License 60/4572).

At first capture, each wood mouse received a unique passive induced transponder tag injected subcutaneously in the scruff (AVID FriendChip), which allowed individual identification of mice at subsequent captures. Furthermore, all mice at first capture were randomly selected to receive either a single oral dose of a combination of anthelmintic drugs: ivermectin (which removes adult worms) at 100 mg/kg and pyranetl palmoate (which removes larval worms) at 9.4 mg/kg (Wahid et al. 1989; Wahid and Behnke 1993; Babayan et al. 2018a; Clerc et al. 2019a), or an equal volume of water as a control. For each mouse at each capture, we recorded the mouse’s trap location, sex, reproductive status (females: perforated vagina, pregnant, or lactating, males: non-reproductive, testes descended, or scrotal), body weight, and length. Fecal samples were collected from pre-sterilized traps each time an animal was captured, and then stored in 10% buffered formalin at 4°C until analysis (Dryden et al. 2005). *Heligmosomoides polygyrus* and *E. hungaryensis* infection (infected/uninfected) and burdens (number of eggs/oocysts per 1 g of feces OPG/EPG) were determined by counting parasite transmission stages (eggs/oocysts) after salt floatation using a microscope at either 10× or 40× magnification (see Knowles et al. 2013).

Mice were sacrificed ∼2-weeks after initial capture and treatment (anthelmintic or control), as previous work with wood mice has shown that anthelmintic drug treatments reduce *H. polygyrus* burdens for only about 10–16 days (Knowles et al. 2013; Clerc et al. 2019a). Spleen and MLNs were collected and stored in sterile RPMI media containing 1% Pen/Strep antibiotics for use in the TNF-α assay. We also collected the digestive tract of each mouse, separated into small intestine, cecum, and colon, to assess burdens of adult gastrointestinal parasites via dissection. The eyes of each mouse were also collected to use dried eye lens weight (milligrams) as a measurement of host age. This metric has been shown to be a reliable method for quantifying host age on a continuous scale, where older hosts have heavier eye lenses compared to younger hosts (Morris 1972; Hardy et al. 1983). Both eyes were stored in 10% formalin at 4°C for at least 4 weeks. After separating the eye lenses from the surrounding tissue, they were dried at 56°C overnight and weighed in pairs to the nearest milligram.

### Immunological methods

To assess the ability of an individual mouse to respond to infection at both the systemic and local levels, we measured pro-inflammatory cytokine TNF-α production by spleen (systemic) and MLN (local) cells *in vitro*. Cells were stimulated with a panel of TLR agonists known to induce production of TNF-α (Akira et al. 2006) including in wild mice (Bradley and Jackson 2008). The agonists used were zymosan (cell surface fungal ligand, TLR2), endosomal oligonucleotide ODN2006 (ODN, TLR9), cell surface *Escherichia coli* K12 lipopolysaccharide (LPS, TLR4), and heat-killed *Listeria monocytogenes* (HKLM, TLR2; all acquired from InvivoGen, San Diego, CA, USA). TNF-α concentration was then measured by sandwich Enzyme-Linked Immunosorbent Assay (ELISA), a previously described, successful method in wild wood mice (Jackson et al. 2009).

Spleen and MLN samples were brought into the lab for cell culture on the same day as collection (tissues in media were kept on ice during transport until used in cell culture). Under sterile conditions, tissues were passed through 70 µm nylon mesh into RPMI media containing heat-inactivated fetal calf serum (10% final volume) and 1% Pen/Strep antibiotics. Cell suspensions were spun down at 1200 rpm, supernatant discarded, and resuspended in fresh media (5 mL for spleen cells, 1 mL for MLN cells). Before resuspension, spleen cells were incubated with red blood cell (RBC) lysis buffer for 3 min, inactivated with 10 mL of RPMI media. This suspension was spun down at 1200 rpm, supernatant discarded, and cells resuspended with 5 mL media. If all RBCs were not lysed, this previous step was repeated a second time. Cell concentrations for each sample were then counted (Cellometer Auto T4 Bright Field Cell Counter, Nexcelom Bioscience) and resuspended to 1 × 10^7^ cells/mL for spleen cell suspensions, 5 × 10^6^ cells/mL for MLN cell suspensions. Different final cell concentrations were used for these two tissue types due to fewer cells available from MLN than spleens; this difference was accounted for in the statistical analyses by analyzing results from each tissue type separately (see below).

Cells from the spleen and MLN were then plated in 96-round bottom well plates for stimulation. Final volumes for samples from each individual mouse varied and low volume samples from individual mice did not receive all agonists. Controls and TLR agonists were run in triplicate, with three wells of cells receiving either an equal volume of media or agonist suspension (100 µL of cell culture, 100 µL of control or agonist added to each well). Concentrations of each agonist were as follows: Zymosan: 20 µg/mL, ODN: 10 µg/mL, LPS: 6 µg/mL, HKLM: 6.0e7 µg/mL (from Jackson et al. 2009). Cell cultures were incubated for 24 h at 37°C (5% CO_2_). Subsequently, plates were spun at 1200 rpm for 2 min and supernatant harvested for use in TNF-α ELISA.

Concentrations of TNF-α produced during cell culture were measured using a mouse sandwich ELISA according to the manufacturer’s instructions (DuoSet ELISA, Mouse TNF-α, R&D Systems). Pooled cell cultures from each mouse for each agonist were used in the ELISA to reduce variation among wells. Each individual mouse and agonist combination was run in triplicate. Plate absorbance was read at 450 nm, the peak wavelength for the plate’s indicator of cytokine concentration, and well absorbance converted to TNF-α concentration (pg/mL) based on the standard curve on each plate. As stated in Jackson et al. (2009), it is likely that affinity of *A. sylvaticus* to the TNF-α antibodies in these ELISA kits differs from *Mus musculus*, but the results should still accurately capture variation in this marker of inflammation among individuals of this species.

### Statistical analysis

All analyses were performed in R (R Core Team 2018), version 3.5.1. Data on parasite infection and burden and *in vitro* inflammatory cytokine concentrations (pg/mL) were analyzed using general linear mixed models (GLMM) in the glmmADMB package. Initial data on parasite infection burden were determined measuring EPG/OPG of *H. polygyrus* or *E. hungaryensis* from samples collected at first capture, prior to the drug (or control) dosing (“Methods” section, see Clerc et al. 2018). Final measures of parasite infection were (1) final adult *H. polygyrus* burdens within the gut (number of adult worms) and (2) final *E. hungaryensis* infection (presence [1]/absence [0] of oocysts in feces). Analyses were performed using mean TNF-α concentrations for each individual and TLR agonist combination. Cytokine concentrations were log-transformed (log10 [1+TNF-α concentration]) to improve normality (Gaussian distribution in GLMM).

We measured the production of TNF-α from splenocytes as an indicator of the systemic immune response and MLN cells as an indicator of the local immune response. There was variation in mean concentration of TNF-α produced *in vitro* in response to stimulation with different agonists, however, we analyzed TNF-α concentrations from all control and stimulated wells together, as sample size did not permit analyzing data from each agonist individually or inclusion of the agonist type as predictor variable in models. While this did not allow us to evaluate how a host may respond to a specific kind of infection (via the response to a single agonist), it did allow us to capture variation in TNF-α among individuals in response to anthelmintic treatment and simulate the complex innate immune stimuli that could be experienced by these mice (Jackson et al. 2009).

We included the following variables in each model as possible drivers of variation in the marker of inflammation (TNF-α production in response to the panel of agonists): anthelmintic treatment (treated/control), total *H. polygyrus* adult worm burden, *E. hungaryensis* infection (0/1), host sex (M/F), reproductive condition as determined by the presence of descended testes in males, and females that are visible pregnant or lactating (0/1), eye lens mass (proxy for age), and relevant biological interactions between treatment and other variables (Supplementary Table S1). We also separately analyzed initial *H. polygyrus* and *E. hungaryensis* infection burdens (EPG/OPG from first capture) as possible predictors of final TNF-α production (Supplementary Table S1). We performed model reduction of all full models using maximum likelihood ratios to compare the full model to reduced models using the “anova” function in the glmmADMB package. Interactions followed by main effects were not retained in the final model if the maximum likelihood ratio test had a *P* >0.05, indicating that these terms did not significantly improve model fit. For TNF-α concentrations, random effects of mouse ID nested within ELISA plate, and TLR agonist were included in each model, to account for multiple measures for each individual mouse and variation in stimulation between TLR agonists. A summary of the final GLMMs for parasite burdens and TNF-α concentrations can be found in Table 1.

**Table 1:** Summary of final GLMMs analyzing parasite burdens and TNF-α production

	Final model	Variables	*z*-value	*P*-value
Hypothesis: Males will have higher initial *H. polygyrus* EPG than females.	Initial *H. polygyrus* EPG ∼ Trt + Sex + Trt × Sex	Treatment	−0.17	0.8663
Hypothesis supported? Yes		Sex	3.17	0.0015**
		Treatment × Sex	0.88	0.3808
	Initial *E. hungaryensis* OPG ∼ Sex	Sex	−1.34	0.18
Hypothesis: Drug treatment will lower *H. polygyrus burden*, increase *E. hungaryensis* oocyst shedding.	Final *H. polygyrus* burden ∼ Trt + Reproductive Condition	Treatment	−5.34	<0.0001***
Hypothesis supported? Yes		Reproductive Condition	1.76	0.0783
Hypothesis: Reproductively active hosts will have higher parasite burdens compared to non-reproductively active hosts.	Final *E. hungaryensis* OPG ∼ Treatment	Treatment	2.04	0.042*
Hypothesis supported? Yes (*H. polygyrus* only)				
Hypothesis: Treated males will have higher TNF-α concentrations than control males.	log10 (1 + TNF Concentration Spleen) ∼ Trt +Sex + Final *H. polygyrus* burden + Trt * Sex	Final *H. polygyrus* burden	−1.59	0.111
Hypothesis supported? No (spleen and MLN)		Treatment	−2.55	0.011*
		Sex	0.95	0.34
		Treatment × Sex	1.82	0.069
		Final *H. polygyrus* × Treatment	2.36	0.018*
Hypothesis: Older hosts will have higher TNF-α concentrations compared to young hosts. Hypothesis supported? No (MLN only)	log10 (1 + TNF Concentration MLN) ∼ Sex + Reproductive Condition + Age (eye lens mass) + Treatment + Final *H. polygyrus* burden + Trt × Age + Trt × Final *H. polygyrus* burden	Treatment	2.48	0.013*
		Sex	2.31	0.021*
		Reproductive Condition	−0.89	0.379
		Age (Eye lens mass)	−0.96	0.338
		Final *H. polygyrus* burden	1.46	0.144
		Age × Treatment	−2.72	0.006**
		Treatment × *H. polygyrus* burden	3.4	0.0006**
		Treatment × Reproductive Condition	−1.36	0.174

Results are organized according to hypotheses presented and if the results support these hypotheses. Global (full) models, including main and random effects, are presented in Supplementary Table S1.

## Results

### Impact of anthelmintic treatment and host characteristics on the gastrointestinal parasite community

**Fig. 1:**
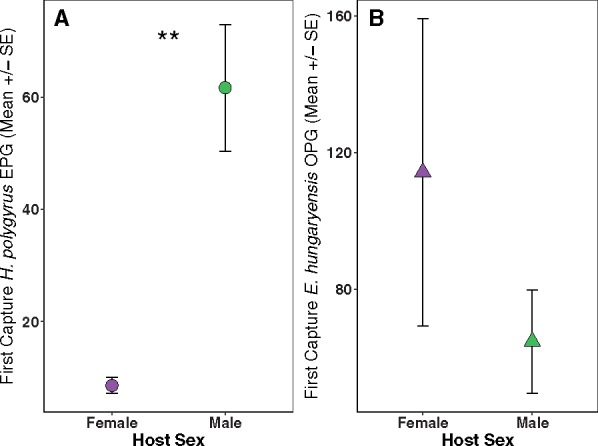
At first capture prior to anthelmintic treatment (**A**) male wood mice shed more *H. polygyrus* eggs per gram feces (EPG; circles), (**B**) but there was no significant difference in *E. hungaryensis* oocyst shedding (OPG feces; triangles).

A total of 148 wood mice were captured during the two replicate field experiments (2014 and 2015). The overall recapture rate (percentage of hosts recaptured at least once) was over 90% for this population; 60% of control hosts, and 57% of drug-treated hosts were captured during the 12-18 day period post-treatment. TNF-α concentrations were measured from 19 mice in Fall 2014 and 30 mice in Summer 2015 (for a summary of demographics and sample size, see Supplementary Table S2). Wood mice were commonly infected with gastrointestinal parasites: 86% (42/49) with the nematode *H. polygyrus*, while 57% (29/49) were infected with the coccidian protozoan *E. hungaryensis*; 14% (7/49) hosts were coinfected when terminally sampled. There were no initial differences in *H. polygyrus* or *E. hungaryensis* infection intensity (EPG/OPG) between mice randomly allocated to anthelmintic treatment or control at first capture (first-capture parasite EPG/OPG: *H. polygyrus*: *z* = 0.47, *P* = 0.64; *E. hungaryensis*: *z* = −0.26, *P* = 0.796). Male mice, however, showed significantly higher initial *H. polygyrus* egg shedding than females at first capture (*z* = 3.17, *P* = 0.0015, Fig. 1A), but no such sex difference was found in initial *E. hungaryensis* oocyst shedding (Fig. 1B).

**Fig. 2:**
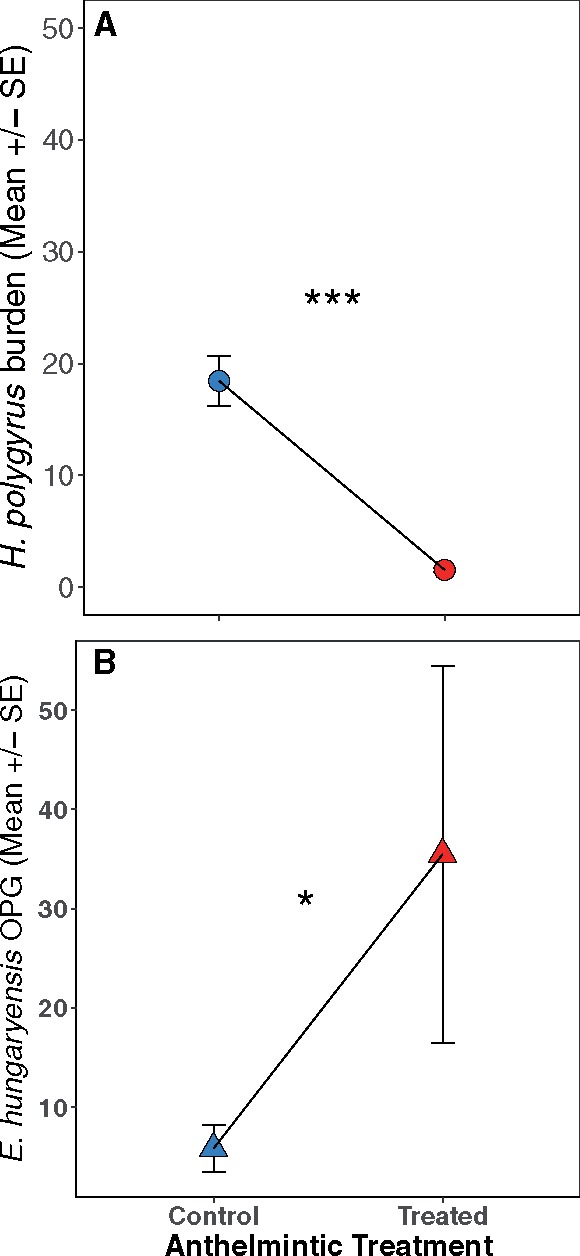
(**A**) Anthelmintic treatment significantly reduced the burden of adult *H. polygyrus* worms (circles) in treated wood mice. (**B**) Anthelmintic treated mice also had a significant increase in the non-target coccidian parasite *E. hungaryensis* fecal OPG (triangles), which suggests evidence of a competition between these coinfecting gastrointestinal parasites.

**Fig. 3:**
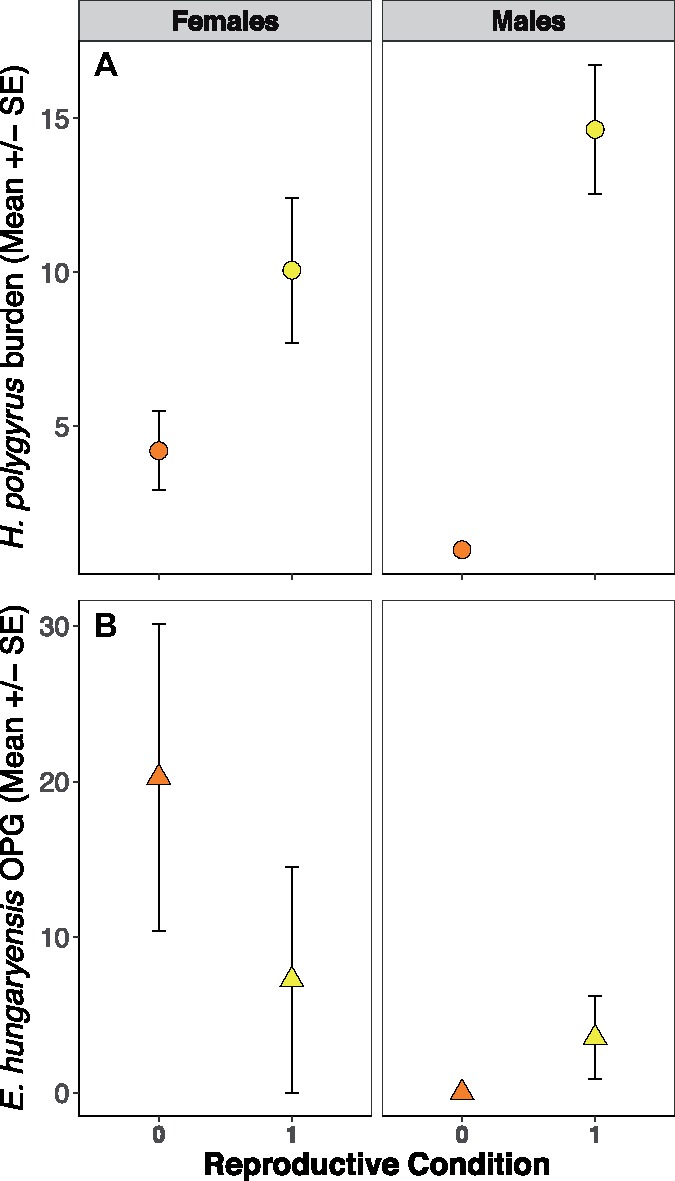
(**A**) Reproductively active (noted by “1”) mice had higher H. polygyrus worm burdens in their gut (circles) compared to non-reproductive mice (noted by “0”), and (**B**) reproductive females had reduced *E. hungaryensis* OPG (triangles). There was no difference in *E. hungaryensis* oocyst shedding with reproductive status in male mice. All mice, control and treated, included here.

We found that anthelmintic treatment significantly reduced *H. polygyrus* adult worm counts when hosts were captured 2 weeks post-treatment (*z* = −5.34, *P* < 0.0001, Fig. 2A). While no mice were completely cleared of *H. polygyrus* worms, treated mice had a nearly 20-fold decrease in worm burdens compared to control mice. Anthelmintic treated mice also had significantly higher *E. hungaryensis* oocyst shedding (OPG, *z* = 2.04, *P* = 0.042, Fig. 2B), with OPGs being four-fold higher than control mice, which is consistent with our previous results (Knowles et al. 2013; Clerc et al. 2019a, 2019b). Even though we had limited statistical power to test for the effect of reproductive status and sex on parasite burdens following anthelmintic treatment (only one non-reproductive male in the dataset, see Supplementary Table S2), we found a trend toward a positive effect of reproductive activity on worm burdens. The *H. polygyrus* burdens of reproductively active females were on average double that of non-reproductive females, while reproductively active males had four times more adult *H. polygyrus* worms in their gut compared to non-reproductive mice (*z* = 1.76, *P* = 0.078, Fig. 3A), however sex as a main effect was not retained in the final model. There were no statistically significant differences in *E. hungaryensis* burden between reproductive and non-reproductive mice (Fig. 3B).

### TNF-α production

Cell cultures stimulated with TLR agonists did produce more TNF-α than control cultures (*z* = 10.78, *P* < 0.0001; control: mean log10 [1 + TNF-α concentration] 2.05 ± 0.170, stimulated: mean log10 [1 + TNF-α concentration] 4.05 ± 0.099; Supplementary Fig. S1).

**Fig. 4:**
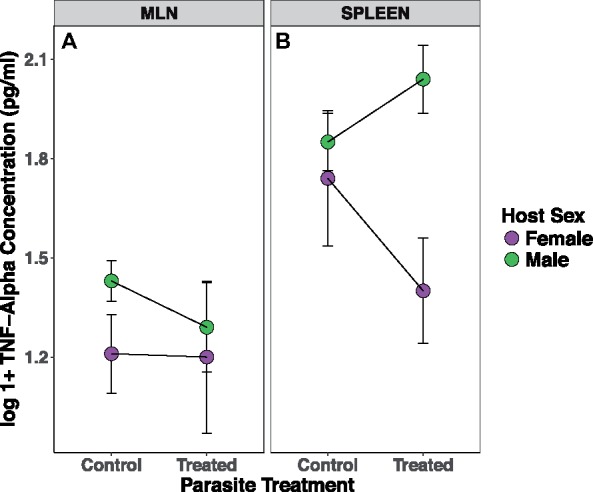
There was a significant effect of both host sex and anthelmintic treatment on TNF-α production at both local (**A**) and systemic (**B**) scales. There was a significant interaction between these variables impacting systemic TNF-α production (B): anthelmintic treated (day ∼14), male mice had higher in TNF-α production *in vitro* (measured by ELISA in pg/ml) at the systemic scale (spleen cell cultures), while treated females showed a decrease in this pro-inflammatory cytokine systemically (mean ± 1 SE shown).

TNF-α production was significantly different between systemic (spleen) and the local (MLN) scales, with systemic TNF-α levels being consistently higher than those measured locally (Spleen: 4.2 ± 0.14, MLN: 3.1 ± 0.12, *z* = 5.63, *P* < 0.0001; Fig. 4), likely due to the difference in TNF-α producing cell concentrations between tissues. While there was a correlation between mean TNF-α concentrations measured from spleen and MLN cells, only 7% of the variation in cytokine concentrations in one tissue was explained by concentrations in the other (*P* = 0.068, adjusted *R*^2^ = 0.072; Supplementary Fig. S2). Therefore, we analyzed local and systemic TNF-α production separately in our subsequent analyses. In addition, we found no significant difference in TNF-α concentrations between field experimental replicates (spleen: *z* = 0.74, *P* = 0.46, MLN: *z* = −0.01, *P* = 0.99), so data were pooled from both years for analysis.

At the systemic level, we found a significant effect of anthelmintic treatment on TNF-α production (*z* = −2.55, *P* = 0.011) and an interaction between treatment and host sex (sex × treatment, *z* = 1.82, *P* = 0.069, Fig. 4B); systemic TNF-α in treated males was ∼50% higher than those from control males. The opposite pattern appeared in females, where treated females showed ∼50% lower systemic TNF-α concentrations than control females.

**Fig. 5:**
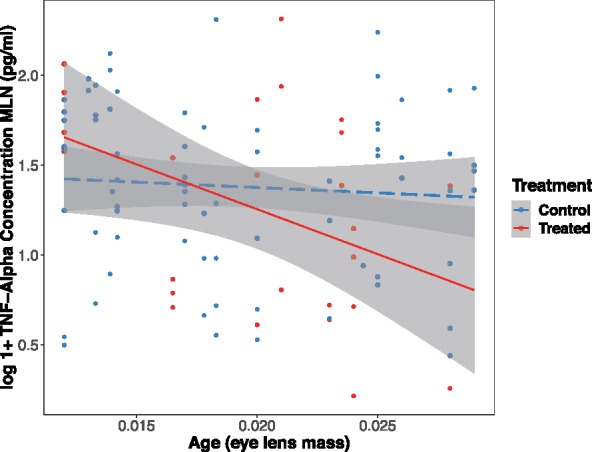
Local TNF-α production (measured by ELISA in pg/mL) measured from MLN cells *in vivo* was reduced in older, treated mice (solid line), while we found no difference in TNF-α production across ages in control mice (dashed line; shaded areas represent standard error of linear regressions for each group).

At the local scale, we also found a main effect of sex on local TNF-α production, with males producing ∼25% more TNF-α than females (*z* = 2.31, *P* = 0.021; Fig. 4A). At the local scale, we found a significant interaction between anthelmintic treatment and host age (age × treatment, *z* = −2.72, *P* = 0.006, Fig. 5); lower production of local TNF-α was found in older, treated mice, while there was no effect of age on local TNF-α production in control mice. These interactions between host characteristics and anthelmintic treatment suggest that hosts are impacted differently by the reduction in *H. polygyrus* burden depending on their age or sex (see Clerc et al. 2019a). There was no relationship between pre-treatment *H. polygyrus* or *E. hungaryensis* burdens and post-treatment TNF-α production at either scale.

## Discussion

In this study, we used targeted drug treatments to reduce *H. polygyrus* burdens in wild, demographically heterogeneous wood mice to determine how single and multiple infections with gastrointestinal parasites impacted the host immune response at two within-host scales. We found that anthelmintic treatment reduced burdens of the target parasite, *H. polygyrus*, by 20-fold 14 days after treatment (Fig. 2A). This reduction in *H. polygyrus* burden in turn led to a four-fold increase in oocyst shedding of the non-target parasite *E. hungaryensis* (Fig. 2B). Furthermore, using laboratory mouse reagents, we successfully measured levels of TNF-α production in spleen and MLN cell cultures, allowing us to use the levels of this important pro-inflammatory cytokine as a marker of inflammation and immune reactivity at different scales. We found that hosts responded differently to anthelmintic treatment depending on demographic factors (age and sex) and importantly, that the relationships between TNF-α production and infection status or demographics differed between the systemic (spleen) and local (MLN) scale. These results have implications for predicting the outcome of parasite coinfection, host health, and parasite transmission dynamics. Perhaps more importantly, however, they increase our understanding of how host demography affects variation in immune markers, and the immune response more generally, depending on the scale at which they are measured.

Overall, we found that male mice had higher initial *H. polygyrus* infection burdens, as measured by egg shedding. Both local and systemic *in vitro* production of TNF-α also varied by host sex and anthelmintic treatment, where males had higher levels of this marker of inflammation than females. In addition, there was an interaction between host sex and drug treatment on systemic inflammation, where treated males showed higher systemic TNF-α production compared to control mice, while systemic TNF-α was lower in treated female mice. This was counter to our predictions, which were that males would demonstrate less immune activation than females, in line with the immunocompetence handicap hypothesis (Folstad and Karter 1992). However, a recent meta-analysis found that TNF-α was one of only a few immune markers significantly increased in male mammals compared to females (Kelly et al. 2018). Our results suggest that males may maintain higher production of inflammatory cytokines following removal of adult *H. polygyrus* worms, which are known to stimulate an anti-inflammatory immune response, potentially leading to increased protection from subsequent infections (Lindstrom et al. 2004; Rynkiewicz et al. 2013; Parker et al. 2017). However, further research is needed to elucidate how this may impact costs of immunity, especially since we were unable to measure the immune reactivity before anthelmintic treatment, as the assay requires culling of animals.

We found that local TNF-α production varied with host age in response to anthelmintic treatment, with older, treated mice having lower TNF-α production in the MLN than younger treated mice, however, there was no effect of age on local TNF-α concentrations among untreated hosts (Fig. 5). In contrast, we previously found that *H. polygyrus*-specific IgG1 antibody titers increased with host age, possibly in response to repeated exposure throughout a host’s lifetime (Clerc et al. 2019a). We predicted that older hosts would have higher levels of pro-inflammatory cytokines following anthelmintic treatment, as older hosts often have higher parasite burdens and/or richness compared to younger hosts (Poulin 1996), as well as often experience chronic elevation of inflammatory markers due to immunosenescence (Martin et al. 2006; Cevenini et al. 2013). While the latter has been shown in wild populations of Soay sheep (Nussey et al. 2009), the processes related to ageing have not been extensively studied in wild mice, and we do not know at what age such effects would become detectable. Interestingly, we only saw variation in our marker of inflammation across different ages after anthelmintic treatment and subsequent *H. polygyrus* burden reduction. Chronic helminth infection may mask the effects of ageing on the wood mouse immune system, therefore making it difficult to see changes in immune strategy with age without removing this dominant gut parasite. The results presented here, in conjunction with those of Clerc et al. (2019a), show how measuring markers of host immunity in response to an experimental manipulation is a robust method for investigating the ecological and evolutionary implications of parasite infection.

The finite amount of resources available to an individual can lead to trade-offs between many important functions, such as growth, reproduction, and the immune response. Often, these trade-offs are context-dependent, based on season, food availability or nutritional content, reproductive state, age or parasite coinfection (Nelson 2004; French et al. 2007; Pedersen and Greives 2008). Several studies have shown that reproduction can redirect host resources away from the immune system in both males and females, via hormonal changes or seasonal changes in physiology (Nelson et al. 2002; Greenman et al. 2005). In our data, there was a slight trend toward reproductively active mice having higher final *H. polygyrus* burdens compared to non-reproductive mice, which also depended on anthelmintic treatment status (Fig. 3). Males also showed increased levels of both local and systemic TNF-α production compared to females, which was again impacted by anthelmintic treatment (Fig. 4). This is consistent with what has been observed in captive zebra finches given an experimental immune challenge, where males always showed higher induced inflammation compared to females, even though both sexes had lower general inflammation when breeding compared to a non-breeding state (Love et al. 2008). Male wood mice had significantly higher initial *H. polygyrus* burdens compared to females, and also shed fewer *E. hungaryensis* oocysts. Recently it has also been found that age, *H. polygyrus* burden before anthelmintic treatment, and *E. hungaryensis* co-infection had significant effects on *H. polygyrus* specific antibody responses in this population of wild wood mice (Clerc et al. 2018, 2019a), so a combination of physiological- and parasite-driven challenges may be impacting the pro-inflammatory marker (TNF-α) measured here. Low sample sizes hindered our ability to analyze the effects of reproductive condition on local and systemic inflammation, but this is clearly a physiological process that can have significant impacts on allocation of within-host resources and immune response and is an avenue for future research.

By measuring the same marker of inflammation at two different within-host scales, we found that reducing *H. polygyrus* burden through anthelmintic treatment affected the host immune response differently at the local versus systemic scales. Many studies have found that gastrointestinal helminths can have significant effects on a host’s immune profile at the systemic scale (spleen/blood) by shifting the immune response toward an anti-inflammatory Th2/T_reg_ type profile (McKay 2006; Maizels et al. 2009; Allen and Maizels 2011). However, how a host responds to the parasite at the site of infection, in our case the gastrointestinal tract, is also important for understanding the interaction between host and parasite and possible effects on locally-coinfecting parasites. Local inflammation has been measured by proxy using the MLNs surrounding the gut (Eberhardson et al. 2008), however measuring immune responsiveness in these tissues is difficult in rodents as it is a terminal procedure (Munoz et al. 2005). There was a correlation between the TNF-α concentrations produced from cells of each tissue within a host, but very little variation was explained (*R*^2^ = 0.07), so it seems likely that the host characteristics we used in our analyses are more informative in explaining the local and systemic production of this pro-inflammatory cytokine.

TNF-α production by spleen and MLN cells varied significantly due to different aspects of host demography as well as anthelmintic treatment, as was seen by the impacts of age and treatment on local TNF-α concentrations but not systemic concentrations. These complementary, but distinct, measures of the pro-inflammatory response measured at a local and systemic scale allow us to better assess the wood mouse immune response to anthelmintic treatment, and perhaps apply this same approach to understand helminth infections and responses to parasite control measures in other systems. These results also emphasize the importance of rigorously studying the immune system in wild, demographically diverse hosts, to truly understand the ecological and evolutionary context of how hosts respond to immune challenges. In conclusion, considering a hosts’ immune system as uniform when studying within-host parasite interactions does not reflect the reality of individuals living in natural environments. Further research into utilizing the approach of measuring markers of immunity at multiple within-host scales will further aid in better understanding parasite–host interactions in the wild.

## Supplementary Material

icz136_Supplementary_Data
