# Supplementary material for: Variation in Local and Systemic Pro-Inflammatory Immune Markers of Wild Wood Mice after Anthelmintic Treatment
Source: Integr Comp Biol. 2019 Aug 1;59(5):1190–202. doi: 10.1093/icb/icz136 (PMC6863754; doi:10.1093/icb/icz136)
Supplement: icz136_Supplementary_Data [file icz136_supplementary_data.zip › Rynkiewicz Supplemental Figures.docx]

**Supplemental Figure 1.** Cells stimulated with toll-like receptor (TLR) agonists produced significantly higher TNF-α compared to cells that were not stimulated (controls), supporting that these wood mouse cells did respond to in vivo stimulation methods. Concentrations of each agonist (µl/ml) used were based on the methods described in Jackson et al. (2009).

**Supplemental Figure 2.** Correlation between TNF-α concentrations (pg/ml) in splenocyte and mesenteric lymph node (MLN) cells. This correlation was not statistically significant and little variation was explained by a linear regression (p = 0.068, adjusted R^2^= 0.072).
